# Larotrectinib Before Initial Radioactive Iodine Therapy in Pediatric TRK Fusion–Positive Papillary Thyroid Carcinoma: Time to Reconsider the Treatment Paradigm for Distantly Metastatic Disease?

**DOI:** 10.1200/PO.21.00467

**Published:** 2022-04-14

**Authors:** Steven G. Waguespack, Sanjit O. Tewari, Naifa L. Busaidy, Mark E. Zafereo

**Affiliations:** ^1^ Department of Endocrine Neoplasia and Hormonal Disorders, University of Texas MD Anderson Cancer Center, Houston, TX; ^2^ Department of Pediatrics-Patient Care, University of Texas MD Anderson Cancer Center, Houston, TX; ^3^ Department of Nuclear Medicine, University of Texas MD Anderson Cancer Center, Houston, TX; ^4^ Department of Head and Neck Surgery, University of Texas MD Anderson Cancer Center, Houston, TX

## Introduction

Papillary thyroid carcinoma (PTC) diagnosed at a pediatric age (≤ 18 years), compared with its adult presentation, is associated with larger primary tumors, more extrathyroidal extension, and an increased prevalence of both locally and distantly metastatic (stage II) disease.^1^ Depending on the series, up to 25% of patients with pediatric PTC will develop pulmonary metastases.^2-6^ Stage II PTC diagnosed during childhood is enriched for oncogenic fusion genes primarily involving *RET*, *NTRK1*, and *NTRK3*.^2,7,8^ Recently, several effective and well-tolerated systemic therapies that selectively target the activated kinases resulting from these fusion genes have received regulatory approval in both children and adults.^9^

Management of pediatric stage II PTC includes thyroidectomy and compartment-focused neck dissection by a high-volume thyroid cancer surgeon followed by treatment with radioactive iodine (RAI).^10^ Rarely are kinase inhibitors needed for advanced disease that has progressed despite prior radioiodine. Traditionally, multiple courses of RAI are given until the patient either achieves cure or reaches a maximum lifetime ^131^I exposure, arbitrarily set at a cumulative dose of 600 mCi (22.2 GBq). However, despite repeated courses of ^131^I, a complete response is found in only 0%-22% of children with pulmonary metastatic disease.^2-6^ Furthermore, RAI can be associated with side effects such as salivary gland damage, pulmonary fibrosis, and an increased risk of second primary malignancies.^11-14^ Fortunately, stage II PTC is indolent in most pediatric patients and long-term survival is the norm.^1,2^

Because of its low cure rate and the potential for late effects arising from repeated courses of high-dose RAI, stage II PTC in children is a challenging disease to treat. Better therapeutic approaches are needed that might enhance the cure rates while minimizing the potential consequences of repeated radioiodine doses. Herein, we present an adolescent with a TRK fusion–positive PTC metastatic to the lungs who was treated using a neoadjuvant systemic approach with the selective TRK inhibitor larotrectinib before the initial dose of RAI.

## Case Report

**FIG 1.**
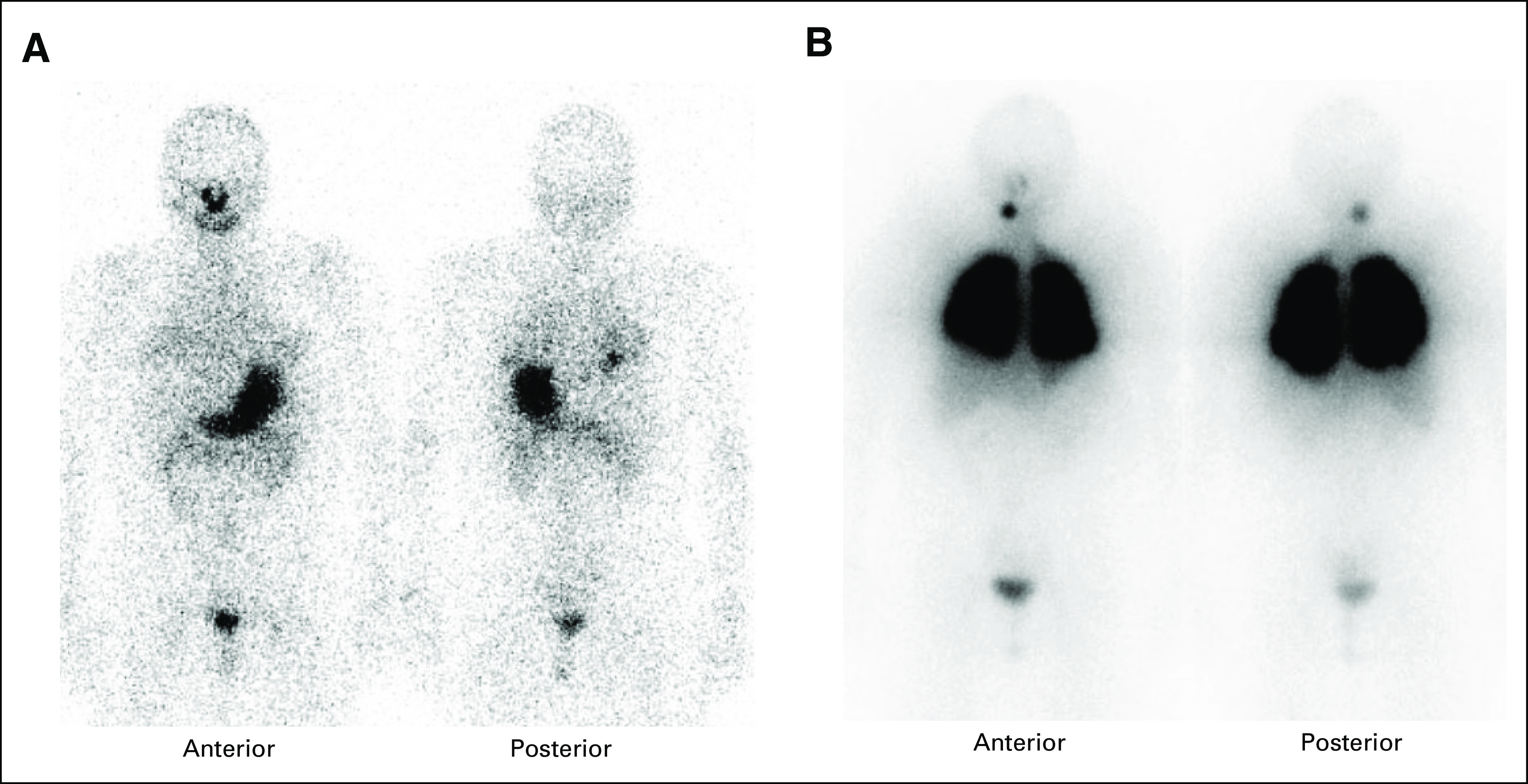
Anterior and posterior whole-body planar images from diagnostic radioactive iodine scans before and after larotrectinib therapy. (A) Hypothyroid diagnostic ^123^I thyroid scan, before larotrectinib, showing no clear uptake in the neck and faint uptake throughout the pulmonary metastatic disease. The calculated uptake in the lungs was 10.2%. (B) Diagnostic ^131^I thyroid scan after recombinant human TSH showed significantly increased uptake in all the pulmonary metastatic disease (26.6% of the ingested dose) and uptake in the right superior thyroid bed, possibly correlating with residual disease left on the right recurrent laryngeal nerve at the cricothyroid joint.

**FIG 2.**
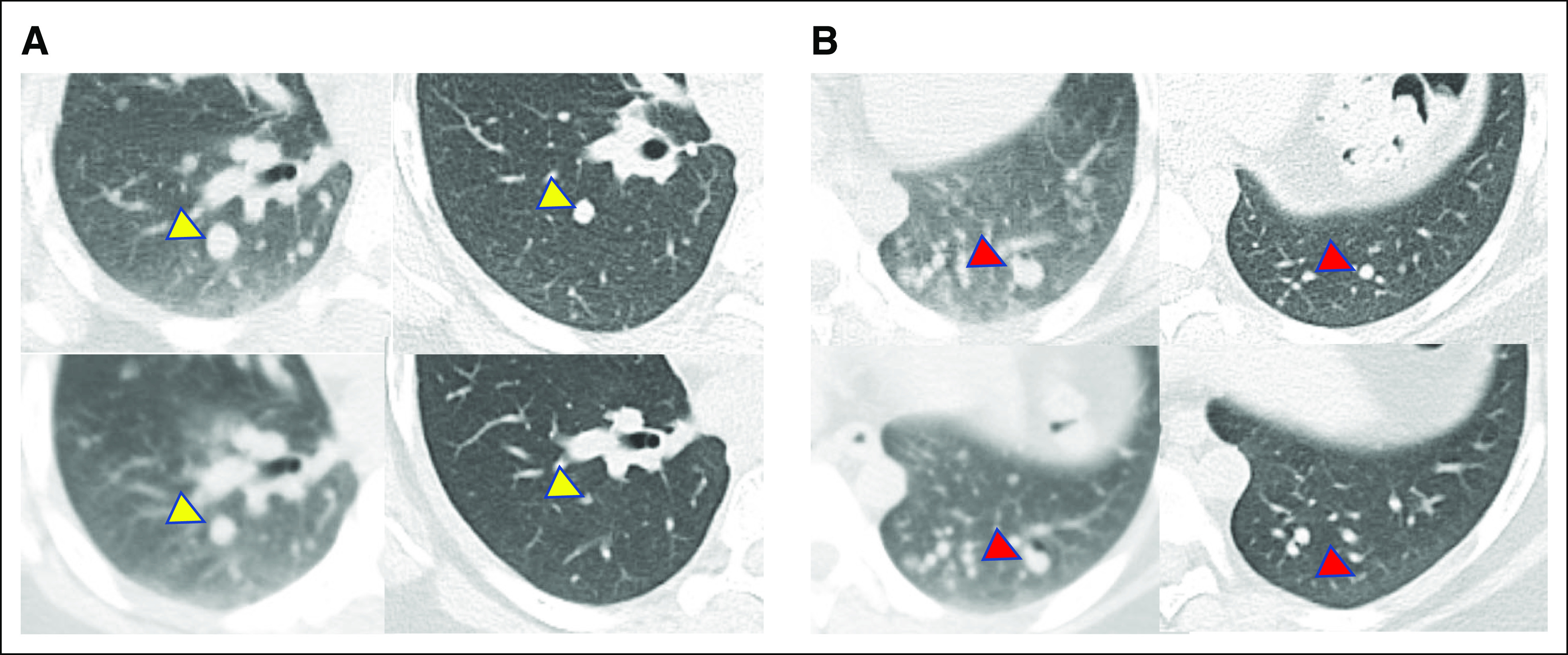
CT images of the two largest pulmonary metastases. (A) A right lower lobe nodule (yellow arrowhead) measured 11 mm at baseline before larotrectinib. The best response to larotrectinib was a 36% decrease after 3 months on therapy, and 1 year after RAI, there was a 67% decrease compared with the scan at the time of RAI and a 73% decrease compared with baseline. (B) A left lower lobe nodule (red arrowhead) measured 10 mm at baseline before larotrectinib. The best response to larotrectinib was a 50% decrease after 3 months on therapy, and 1 year after RAI, there was a 63% decrease compared with the scan at the time of RAI and a 70% decrease compared with baseline. Per RECIST v1.1, the patient's best response to larotrectinib was a partial response (–43%) at 3 months. After RAI, the response in the two target lesions was –65% compared with the scan at the time of RAI therapy and –71% compared with baseline before larotrectinib. For each grouping of CT images, top left = baseline before larotrectinib and at the time of the initial diagnostic SPECT-CT, top right = CT after 3 months of larotrectinib, bottom left = at the time of second diagnostic SPECT-CT after therapy with larotrectinib, and bottom right = CT 1 year after neoadjuvant larotrectinib and 98.7 mCi (3.7 GBq) ^131^I. CT, computed tomography; RAI, radioactive iodine; SPECT, single-photon emission computed tomography.

**FIG 3.**
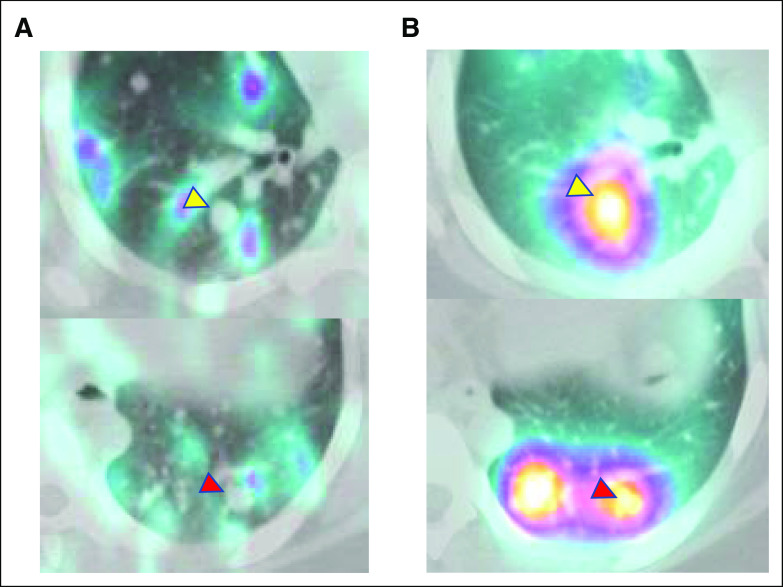
Single-photon emission computed tomography/computed tomography images of the two largest pulmonary metastases at the time of the diagnostic radioactive iodine scan (A) before and (B) after larotrectinib therapy. Right lower lobe (yellow arrowhead) and left lower lobe (red arrowhead) metastases were initially not iodine-avid but demonstrated significantly increased radioiodine uptake after larotrectinib therapy.

The patient is a Hispanic female who presented to our institution at age 15 years for a newly diagnosed PTC. Preoperative staging with neck ultrasonography and contrast-enhanced computed tomography (CT) of the neck and chest identified bulky central and right-sided cervical disease and evidence for bilateral micro- and macronodular pulmonary metastatic diseases. She was treated with total thyroidectomy and comprehensive central and right lateral neck dissections. Final American Joint Committee on Cancer, eighth edition stage group was II (T4aN1bM1). DNA and RNA next-generation sequencing identified a *TPM3-NTRK1* fusion gene, a known oncogenic driver identified in about 8% of TRK fusion–positive thyroid cancers.^15^ The patient proceeded to a hypothyroid ^123^I thyroid scan while following a low iodine diet and with a documented low random urine iodine level (Table 1). This showed no clear cervical uptake and faint uptake in the pulmonary metastases (Fig 1A). The largest lesions measured 11 mm in the right lower lobe and 10 mm in the left lower lobe (Fig 2), both of which demonstrated no evidence of iodine avidity (Fig 3A). Given the minimal pulmonary uptake and because of the significant burden of disease in the lungs, it was recommended that she start systemic therapy with larotrectinib. The goal of therapy was to debulk the tumor medically before initial RAI, anticipating that this would enhance the subsequent response to RAI because of smaller volume disease.

**TABLE 1.**
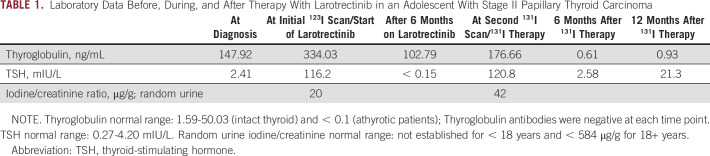
Laboratory Data Before, During, and After Therapy With Larotrectinib in an Adolescent With Stage II Papillary Thyroid Carcinoma

After informed consent, the patient commenced larotrectinib 100 mg by mouth twice a day. After the first dose, she developed headache, lightheadedness, temperature dysregulation in her hands, and tingling in the fingertips. The drug was briefly held and then resumed at the same dose. She developed transient grade 1 leukopenia and grade 1 fatigue but had no other subsequent adverse events. CT imaging showed evidence for a partial response after three months of therapy (Fig 2). After 6 months of therapy, a decision was made to proceed to high-dose ^131^I therapy. Unexpectedly, the recombinant human TSH-stimulated ^131^I thyroid scan showed significantly increased uptake in all the pulmonary metastatic disease, including the previous noniodine-avid disease (Figs 1B and 3B). Indeed, the percent uptake of the ingested dose in the lungs (26.6%) was so high that the administered dose was adjusted downward so as not to place her at undue risk for pulmonary fibrosis. An empiric activity of 98.7 mCi (3.7 GBq) ^131^I was administered approximately 31 hours after the last dose of larotrectinib and the same day as the diagnostic study. She tolerated RAI well with no immediate or delayed side effects. Six months after ^131^I therapy, there was a significant decline in the thyroglobulin level (Table 1). Twelve months after RAI, her (unintended) stimulated thyroglobulin was only 0.93 ng/mL. Chest CT revealed a significant response in all the metastatic nodules (Fig 2), including the disappearance of some of the smaller metastatic deposits.

## Discussion

The treatment of pediatric stage II PTC remains a challenge given its low complete response rates and the inherently higher risks of both surgical and RAI therapies in children. RAI has been the mainstay of treating distantly metastatic disease since the 1940s, and before 2018, there were no systemic therapies approved for use in children with advanced PTC. Recently, it has become better appreciated that most children with stage II disease will have persistent, albeit indolent, disease and that novel therapeutic approaches are needed to improve cure rates. In particular, the long-term sequelae of high doses of RAI may add to the lifelong symptom burden of the patient and increase the risk of secondary malignancies.^11-14^

Larotrectinib is a selective TRK inhibitor that has been approved for the treatment of both pediatric and adult patients with advanced solid tumors that harbor an *NTRK* fusion gene. It is one of two available TRK inhibitors, with the other being entrectinib, which also targets ALK and ROS1.^16^ Treatment with larotrectinib has been proven to be widely effective in both adults and children across various solid malignancies, including PTC, and it is associated with a favorable toxicity profile.^17,18^

Metastatic PTC can lose its ability to concentrate radioiodine, and our increasing understanding of the molecular basis of PTC has given further insight into the mechanisms of RAI-refractory (RAI-R) disease.^19^ The Cancer Genome Atlas was the first study to correlate the molecular basis of PTC with a differentiation score on the basis of gene expression.^20^ *BRAF*^V600E^-like tumors have a lower differentiation score, with reduced expression of genes responsible for iodine uptake and metabolism, whereas *RAS*-like tumors have a higher differentiation score. *NTRK1/3* fusion–positive PTC falls somewhere in between.^7,8,19,20^ The concept of redifferentiation therapy is a relatively new one and currently refers to the inhibition of mitogen-activated protein kinase pathway signaling by a MEK or BRAF kinase inhibitor to improve iodine uptake in metastatic tumors.^21^ Recently, two cases have been published in which larotrectinib was able to redifferentiate RAI-R PTC (Table 2).^7,22^ Although our patient is different in that she was not yet considered RAI-R, her diagnostic thyroid scan before larotrectinib demonstrated minimal RAI uptake in the pulmonary metastases, including the largest lesions that exhibited no appreciable iodine uptake. Larotrectinib therapy was well tolerated and resulted in tumor shrinkage and enhanced iodine uptake in both iodine-avid and nonavid diseases, which allowed the successful administration of a lower dose of ^131^I that, at one year, has resulted in a significant partial response radiographically and a near-complete biochemical response.

**TABLE 2.**
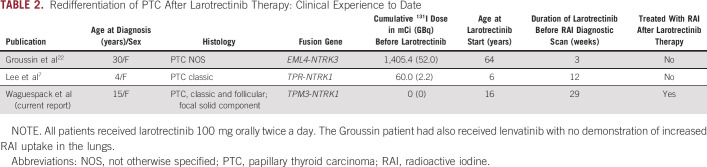
Redifferentiation of PTC After Larotrectinib Therapy: Clinical Experience to Date

Although the current case is an exciting one that suggests a potential shift in the traditional management of distantly metastatic PTC, caution must be undertaken as we learn more about the possible long-term side effects of systemic TRK inhibition, particularly in the context of the concomitant use of RAI. It is also important to better understand the optimal duration of therapy and if it is safe to administer RAI while actively receiving larotrectinib; in the current case, we stopped the medication the day before RAI was administered. It also remains unknown if such an approach would apply to the other commercially available TRK inhibitor entrectinib or to the selective RET inhibitors, selpercatinib and pralsetinib, although recent publications would suggest a similar therapeutic effect with selpercatinib.^7,23^ Ideally, despite the challenges of recruitment and the issues surrounding the assessment of response in nonmeasurable disease, which is typical in young patients with stage II PTC, well-designed clinical trials should be considered to answer these important questions.

In conclusion, to our knowledge, we report the first case of a patient who achieved clinical benefit from initiation of the selective TRK inhibitor larotrectinib in a neoadjuvant fashion before the first dose of RAI. Although the initial goal of therapy was to medically debulk her tumor to allow more effective delivery of ^131^I, the patient's tumor also demonstrated an unexpected and significant increase in RAI avidity (redifferentiation), which we believe improved the therapeutic outcome and permitted the use of a lower administered dose of ^131^I. The time may be upon us to reconsider our treatment paradigm and incorporate selective fusion gene inhibitors such as larotrectinib earlier in the treatment course to maximize the tumor response to RAI in advanced PTC.
